# Bilateral Sudden Hearing Loss in Iron Deficiency Anemia

**DOI:** 10.7759/cureus.54505

**Published:** 2024-02-20

**Authors:** Jin Woo Choi, Sung-Yong Kim, Chang-Hee Kim

**Affiliations:** 1 Department of Radiology, Konkuk University Medical Center, Seoul, KOR; 2 Department of Hematology and Oncology, Konkuk University Medical Center, Seoul, KOR; 3 Department of Otorhinolaryngology, Konkuk University Medical Center, Seoul, KOR

**Keywords:** 3d flair mri, positional nystagmus, iron deficiency anemia, bilateral, sudden hearing loss

## Abstract

The present study describes an unusual case of bilateral sudden hearing loss associated with iron deficiency anemia. Although hematologic disorders such as anemia or leukemia have been reported to be associated with sudden hearing loss, bilateral sudden hearing loss, which was presented as the first manifestation of iron deficiency anemia, has not been reported. A 74-year-old man presented with simultaneous bilateral sudden hearing loss without vertigo. A complete blood count test revealed a hemoglobin level of 6.4 g/dL and a ferritin level of 14.5 mg/mL, indicating iron deficiency anemia. Postcontrast 3D FLAIR MRI showed enhancement of the bilateral cochlea, vestibules, and lateral semicircular and posterior semicircular canals. After treatment, the patient’s hearing loss partially improved.

## Introduction

Sudden hearing loss is diagnosed when an abrupt hearing loss occurs over a period of 72 hours or less. In the United States, five to 20 patients per 100,000 are affected by sudden hearing loss, with approximately 4,000 new cases per year [1]. Most cases are idiopathic, and a variety of causes, including viral infections, vascular disorders, autoimmune diseases, and ototoxic substances, have been proposed for an etiology. The absolute majority of sudden hearing loss cases are unilateral, and unilateral and bilateral sudden hearing loss are known to represent different disease entities. Bilateral conditions are often associated with serious systemic illness and have a higher prevalence of morbidity and mortality [2]. Moreover, bilateral sudden hearing loss is characterized by a more severe degree of hearing loss with a poorer prognosis [3].

Vascular compromise of the inner ear due to reduced blood supply, embolus, thrombosis, or vasospasms, is believed to be associated with the development of sudden hearing loss. Anemia is a condition in which a person has fewer red blood cells than normal and is an important risk factor for cardiovascular and cerebrovascular events [4]. Although a population-based study suggested that there is an association between sudden hearing loss and iron deficiency anemia, bilateral sudden hearing loss associated with iron deficiency anemia has not yet been reported.

In the present study, we present the case of a 74-year-old man with bilateral sudden hearing loss who was newly diagnosed with iron deficiency anemia. We demonstrate the detailed findings of 3D fluid-attenuated inversion recovery (3D FLAIR) internal auditory canal (IAC) MRI and discuss the possible mechanism of bilateral sudden hearing loss.

## Case presentation

A 74-year-old man presented with bilateral sudden hearing loss that had developed three days prior. He did not complain of dizziness, vertigo, or headache. The patient had a past surgical history of thyroid lobectomy due to thyroid papillary carcinoma and was on medication for cardiac arrhythmia and gout. Otoendoscopic examination revealed a normal tympanic membrane on both sides, except for mild calcification, suggesting previous inflammation. Spontaneous nystagmus, gaze-evoked nystagmus, or skew deviation was not observed. Head-shaking nystagmus, vibration-induced nystagmus, or hyperventilation-induced nystagmus was not observed. While other positional nystagmus tests did not provoke positional nystagmus, a Dix-Hallpike test provoked horizontal geotropic positional nystagmus on both sides (Supplemental video 1).

**Video 1:** Video oculography of the patient during a Dix-Hallpike test A Dix-Hallpike test, which is a positioning nystagmus test by putting the patient's head backward about 110 degrees while turning 45 degrees to either side, elicited horizontal geotropic direction-changing positional nystagmus, which means that right-beating nystagmus was observed during the right Dix-Hallpike maneuver and left-beating nystagmus was observed during the left Dix-Hallpike maneuver. Thus, vestibular impairment of the patient was shown to be affected by a position change of the head.

**Figure 1:**
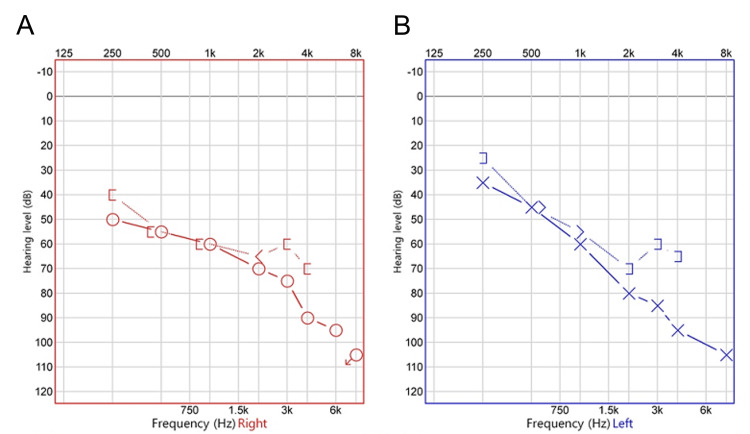
Pure tone audiometry revealed sensorineural type hearing loss on both the right (A) and left (B) sides. (A) Pure tone audiometry of the right ear. X and Y axes represent the tested frequency and hearing threshold level, respectively. Hearing threshold level was 50, 55, 60, 70, and 90 dB at 0.25, 0.5, 1, 2, and 4 kHz, respectively. Thus, this audiogram showed a pattern of downsloping sensorineural hearing loss on the right side. (B) Pure tone audiometry of the left ear. Hearing threshold level was 35, 45, 50, 80, and 95 dB at 0.25, 0.5, 1, 2, and 4 kHz, respectively. Thus, this audiogram showed a pattern of downsloping sensorineural hearing loss on the left side.

Other neurologic examinations, including a cerebellar function test, revealed no abnormalities. Pure tone audiometry showed 65 dB of sensorineural hearing loss with a speech discrimination score of 36% on the right side and 67.5 dB of sensorineural hearing loss with a speech discrimination score of 36% on the left side (Figures 1A, 1B).

**Figure 2:**
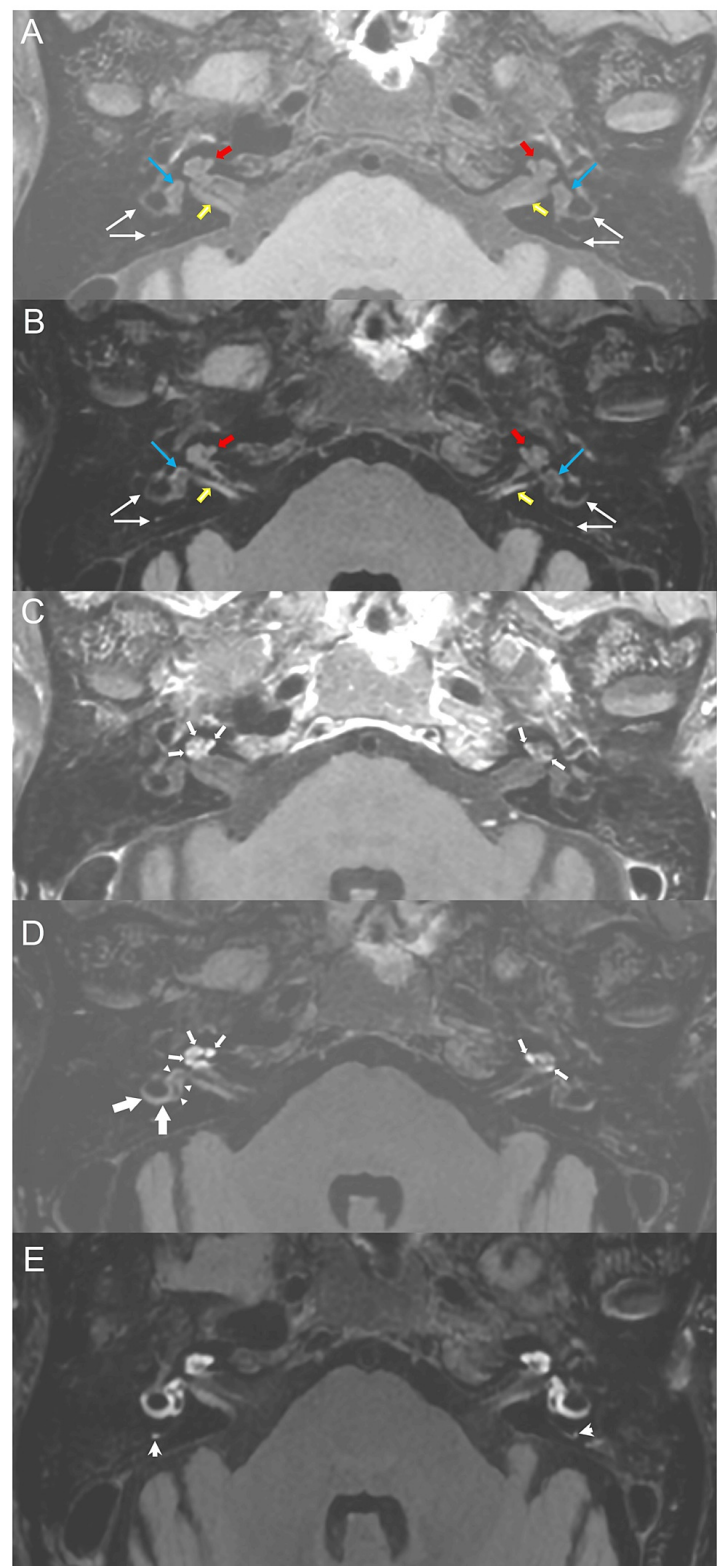
Internal auditory canal magnetic resonance imaging. Precontrast T1-weighted image (A) and precontrast three-dimensional fluid-attenuated inversion recovery (3D FLAIR) images (B) at the level of internal auditory canal (IAC, yellow arrows) show normal signal intensity of the bilateral cochlea (red arrows), vestibules (blue arrows) and semicircular canals (white arrows). After gadolinium-contrast enhancement, enhancement of the bilateral cochlea is noted (small arrows) on postcontrast T1-weighted images (C) and 10-minute-delay 3D FLAIR images (D). Subtle enhancement of the right vestibule (arrowheads) and right lateral semicircular canal (large arrows) is also noted on a 10-minute-delay 3D FLAIR image. Postcontrast, four-hour-delay 3D FLAIR image (E) shows profuse enhancement of the bilateral cochlea, vestibules and lateral semicircular and posterior semicircular canals (open arrow). In this patient, delayed high signal intensity in postcontrast 3D FLAIR image indicates the breakdown of blood-labyrinth barrier on both sides.

A bithermal caloric test showed no unilateral weakness. The result of subjective visual vertical was within normal range, and nystagmus was not elicited by hyperventilation, head shaking or vibration on mastoid bone and sternocleidomastoid muscle. IAC MRI demonstrated normal signal intensity of the bilateral cochlea, vestibules and semicircular canals on precontrast T1-weighted images (Figure 2A) and precontrast 3D FLAIR images (Figure 2B). After gadolinium-contrast enhancement, enhancement of the bilateral cochlea was observed on postcontrast T1-weighted images (Figure 2C) and 10-minute-delay 3D FLAIR images (Figure 2D). Subtle enhancement of the right vestibule and right lateral semicircular canal was also observed on 10-minute-delay 3D FLAIR images (Figure 2D). Postcontrast, four-hour-delay 3D FLAIR images showed profuse enhancement of the bilateral cochlea, vestibules and lateral semicircular and posterior semicircular canals (Figure 2E).

A complete blood count test revealed a hemoglobin level of 6.4 g/dL, a ferritin level of 14.5 mg/mL, a platelet count of 257 x 10^3^/μL, a white blood cell count of 7,120/μL, and a mean corpuscular volume of 61.4 fL, indicating iron deficiency anemia (Table 1).

**Table 1:** Laboratory test results

	Patient’s result	Reference range
Hemoglobin (g/dL)	6.4	13-16.5
Hematocrit (%)	23.7	39-49
Mean corpuscular volume (fL)	61.4	80-98
Ferritin (mg/mL)	14.5	30-400
Platelet (x 10^3^/μL)	257	140-400
White blood cell count (x 10^3^/μL)	7.12	4-10

A gastrointestinal bleeding source was sought because stool hemoglobin was positive, and gastrofibroscopic study showed no abnormality. However, the patient refused to undergo further workup for gastrointestinal bleeding, including colonoscopy.

Under the diagnosis of bilateral sudden hearing loss associated with iron deficiency anemia due to chronic hemorrhage, treatment was commenced immediately. Systemic corticosteroids with simultaneous bilateral intratympanic dexamethasone injection were administered, and treatment for iron deficiency anemia including transfusion and iron supplementation was conducted. Partial recovery of hearing was obtained after treatment and at six weeks after treatment, and pure tone audiometry showed an average threshold of 48 dB with a speech discrimination score of 80% on the right side and an average threshold of 53 dB with a speech discrimination score of 84% on the left side.

## Discussion

This study is, to the best of our knowledge, the first report of bilateral sudden hearing loss associated with iron deficiency anemia. Bilateral cases account for approximately 1%-5% of all patients with sudden hearing loss [3,5-8], and bilateral involvement has been regarded as a “red flag” sign for a more severe underlying condition. The etiology of unilateral sudden hearing loss has not yet been elucidated, and inner ear vascular compromise, viral infection, autoimmune disease, or rupture of Reissner’s membrane has been proposed as a possible etiology [1]. Although the etiology of bilateral sudden hearing loss is also unclear as is unilateral disease, specific underlying conditions such as metastasis from lung cancer, sickle cell anemia, myeloblastic syndrome, macrocytosis, autoimmune inner ear disease, leukemia, meningitis, leptomeningeal carcinomatosis, multiple sclerosis, toxoplasmosis, sarcoidosis, intracranial aneurysm, and infectious mononucleosis, have been reported, unlike unilateral cases, in bilateral sudden hearing loss.

Iron deficiency anemia is the most common type of anemia and is caused by chronic bleeding of the gastrointestinal, uterine, or urinary tract, and insufficient dietary intake or absorption of iron. Based on previous reports that cerebrovascular or cardiovascular diseases are affected by decreased hemoglobin levels and that iron deficiency anemia is an important risk factor for cerebral ischemia [9-11], it is plausible that iron deficiency anemia may have a detrimental effect on inner ear hemodynamics. Another possibility is autoimmune inner ear disorder as an underlying autoimmune-mediated mechanism of bilateral sudden hearing loss based on the observations that iron deficiency anemia is associated with autoimmunity [12,13]. Furthermore, Sun et al. provided experimental evidence that iron deficiency itself can play an important role in the pathogenesis of sudden hearing loss in rats [14]. They observed that approximately 10% of rats raised on a basic iron-deficient diet showed sudden hearing loss to varying extents, from moderate to profound. They also showed a significant reduction in spiral ganglion cells, a rapid impairment of stereocilia of the outer and inner hair cells, and synchronous abnormalities of the iron-containing enzymatic activity in the whole cochleae of rats raised with an iron-deficient diet [14].

Another interesting finding of the present study was that enhancement of the bilateral cochleae, vestibules, and semicircular canals was observed in postcontrast 3D FLAIR MRI, which is, to the best of our knowledge, the first demonstration of bilateral sudden hearing loss. Yoshida et al. showed high signal intensities on precontrast 3D FLAIR IAC MRI, which reflects high protein content or minor hemorrhage in the inner ear fluids, in some patients with unilateral sudden hearing loss, and suggested that this radiologic finding may indicate poor prognosis for hearing recovery [15]. Byun et al. reported that the hearing recovery rate was significantly lower in sudden hearing loss patients with lesion-side laterality on four-hour postcontrast 3D FLAIR MRI images [16]. Our patient showed direction-changing positional nystagmus, which may be in line with the observations of a previous study in patients with sudden hearing loss [17]. Considering the radiologic findings of 3D FLAIR IAC MRI, alteration of protein content in the inner ear fluid may be responsible for the generation of positional nystagmus [18].

## Conclusions

The particularities of this case report are that, first, simultaneous bilateral sudden hearing loss occurred with an initial diagnosis of iron deficiency anemia. Second, profuse enhancement of inner ear organs was observed in postcontrast 3D FLAIR IAC MRI.
